# Ipsilateral Lymphatic and Venous Malformations Affecting the Midface Area

**DOI:** 10.1155/2020/2845035

**Published:** 2020-09-18

**Authors:** Şükran Bekdemir, Ahmet Kaan Gündüz, Ömür Ataoğlu

**Affiliations:** ^1^Ophthalmology Clinic, Polatlı Duatepe State Hospital, Ankara, Turkey; ^2^Department of Ophthalmology, Ankara University Faculty of Medicine, Ankara, Turkey; ^3^Private Mikro-Pat Pathology Laboratory, Ankara, Turkey

## Abstract

A 22-year-old woman presented with progressive swelling of the nasal conjunctiva in the left eye. Anterior segment examination revealed a diffuse cystic appearance to the inferonasal bulbar conjunctiva and plica semilunaris. Anterior segment swept-source optical coherence tomography (OCT) revealed clear hyporeflective spaces demarcated by hyperreflective septae in the affected conjunctiva, consistent with the diagnosis of lymphatic malformation (LM). Magnetic resonance imaging revealed a well circumscribed intraconal mass located inferonasally in the left orbit. Systemic examination revealed a lesion similar to LM on the left hard palate. The left conjunctival mass was excised subtotally. Subsequently, a transconjunctival anterior orbitotomy was performed and the left orbital mass was completely removed intact. Histopathologically, the conjunctival mass was diagnosed as LM and the orbital mass as venous malformation (VM). This case represents a rare coexistence of histopathologically proven conjunctival LM and orbital VM as well as a presumed LM of the hard palate, all 3 lesions occurring in the ipsilateral midface area.

## 1. Introduction

Hemangiomas develop as a result of abnormal changes in angiogenesis that allow overproliferation of vascular tissues. The term “hemangioma” has been superceded by cavernous venous malformation or venous malformation (VM). Several authors have elucidated the interaction between angiogenic and angiostatic forces involved in normal and pathologic processes [1]. Many of the angiogenic markers including fibroblast growth factor (FGF), vascular endothelial growth factor (VEGF), E-selectin, and type IV collagenase are increased during the proliferative growth phase of hemangiomas [2]. It has been speculated that hemodynamic changes cause opening of new channels allowing for budding of the vascular channels into surrounding soft tissues. After sufficient growth of the lesion, cosmetic or visual disturbances arise leading to imaging studies to be performed so that the lesion is diagnosed.

Lymphangiomas (referred to as lymphatic malformations, LM) are collections of lymph vessels filled with serous fluid. The formation of LMs may reflect a failure of lymph ducts to connect with the venous system during embryogenesis, abnormal sequestration of lymphatic structures, or both [3]. Many LMs are initially asymptomatic until an inciting factor causes an increase in the size of the lesion. Growth of the LMs is usually related to upper respiratory tract infections and minor trauma to the periocular region.

We herein report an interesting case demonstrating ipsilateral coexistence of orbital VM, conjunctival LM, and hard palate LM.

## 2. Case Presentation

**Figure 1:**
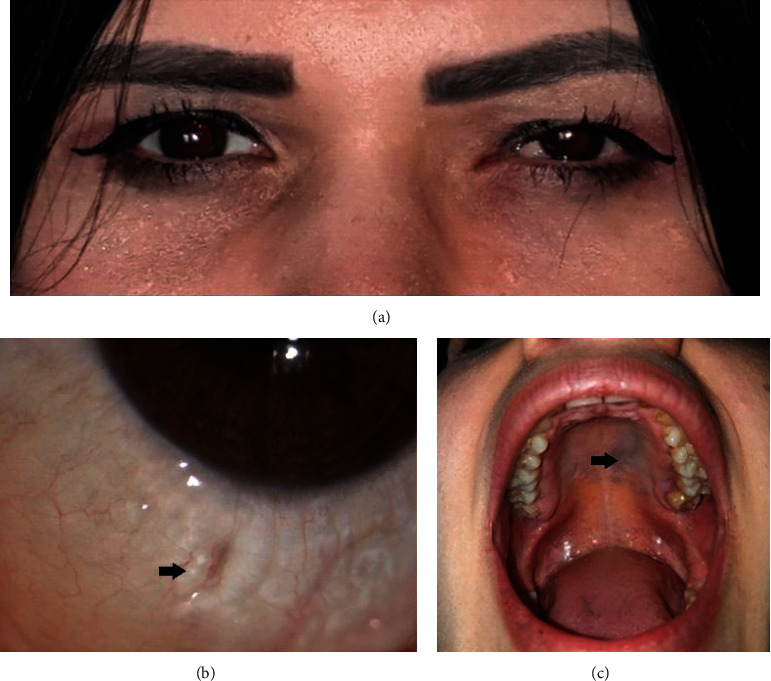
(a) Facial appearance of the patient at presentation showing slight lateral displacement of the left eye. (b) Anterior segment photograph shows lymphatic malformation cysts in the inferonasal conjunctiva (arrow). (c) Lesion similar to lymphatic malformation affecting the left hard palate (arrow).

**Figure 2:**
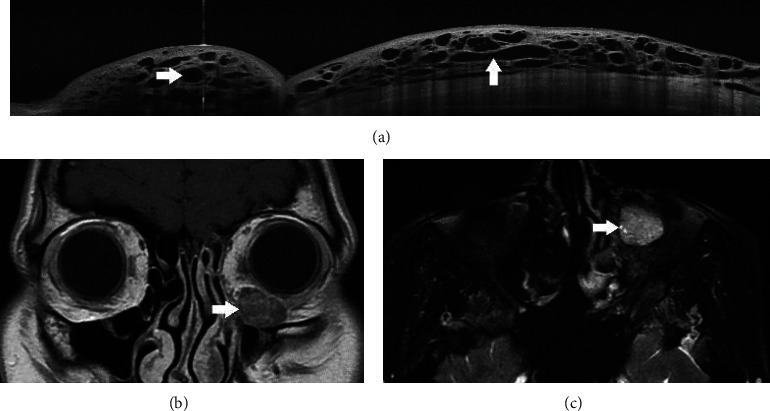
(a) Anterior segment swept source optical coherence tomography of the left eye demonstrates dilated lymphatic channels manifesting as hyporeflective spaces with different sizes demarcated by hyperreflective septae (arrows). (b) T1-weighted coronal MR image shows the inferonasally located intraconal tumor that is isointense with respect to muscle (arrow) in the left orbit. (c) T2-weighted axial MR image shows that the left orbital tumor is hyperintense (arrow).

A 22-year-old woman presented with progressive nasal conjunctival swelling in the left eye of 2-years duration. The patient had no history of systemic illness, ocular disease, or allergy. Thyroid function tests were within normal limits. Family history was negative for any ocular and cranial vascular malformation. Visual acuities at presentation were 20/20 in the right and 20/40 in the left eye. Intraocular pressures were 17 mmHg in both eyes. There was no relative afferent pupil defect bilaterally. Ocular motility was full, and there was no proptosis in either eye. The left eye was mildly displaced laterally although the patient did not complain of double vision (Figure 1(a)). Anterior segment biomicroscopic examination of the left eye revealed that the plica semilunaris and inferonasal bulbar conjunctiva had a multicystic appearance (Figure 1(b)). Anterior segment and fundus examination of the right eye were normal. There was a lesion similar to LM on the patient's left hard palate (Figure 1(c)). Anterior segment swept-source optical coherence tomography (AS-SSOCT) (DRI OCT Triton Plus, Topcon, Tokyo, Japan) showed there was elevation of the plica semilunaris and bulbar conjunctiva comprising variable-sized hyporeflective spaces separated by hyperreflective tissue in the left eye (Figure 2(a)). AS-SSOCT of the right eye revealed normal findings. The patient subsequently underwent orbital and brain magnetic resonance imaging (MRI) which revealed an oval-shaped, intraconal mass located inferonasally in the left orbit.The orbital mass was in the same quadrant as the conjunctival LM. The mass was isointense with respect to the extraocular muscles on T1-weighted images, hyperintense on T2-weighted images, and demonstrated contrast enhancement (Figures 2(b) and 2(c)). There was no vascular malformation affecting the brain and meninges.

**Figure 3:**
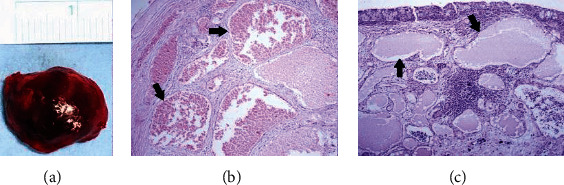
(a) Gross photograph of the excised left orbital tumor showing the reddish well-circumscribed mass measuring approximately 1.5×1.5 cm in base dimensions. (b) Histopathological examination of the orbital tumor reveals enlarged vascular channels with thick walls, the lumens of which are filled with erythrocytes embedded in a fibrous stroma (arrows), consistent with orbital cavernous venous malformation (H.E. ×100). (c) Histopathological examination of the conjunctival lesion shows several ectatic bloodless lymphatic channels, lined with flattened endothelium (arrows). There is prominent lymphoid infiltration and aggregates in the stroma, consistent with the diagnosis of conjunctival lymphatic malformation (H.E. ×100).

**Figure 4:**
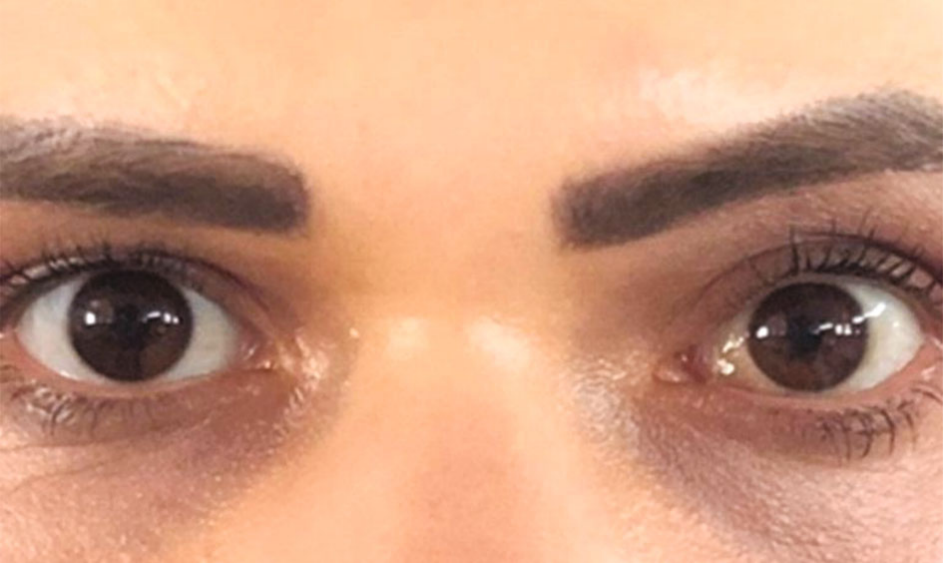
Facial photograph of the patient 3 months after surgery shows excellent outcome with an orthophoric appearance.

Surgical intervention was planned to perform conjunctival biopsy and to remove the orbital tumor. Under general anesthesia, the patient first underwent subtotal excisional biopsy of the left conjunctival mass. Subsequently, an anterior orbitotomy was performed via the inferonasal transconjunctival approach and the orbital tumor was removed in toto with the help of a cryoprobe. The basal diameters of the excised reddish, smooth-edged, capsulated orbital mass were approximately 1.5 × 1.5 cm (Figure 3(a)). Histopathological examination of the orbital mass revealed enlarged vascular channels featuring thick walls with fibrosis, the lumens of which were filled with erythrocytes. The histopathological diagnosis of the orbital mass was established as VM (Figure 3(b)). Histopathological examination of the conjunctival lesion revealed a vascular structure containing lymphatic fluid surrounded by endothelium and conjunctival epithelial cells on the surface, consistent with LM (Figure 3(c)). At 3 months follow-up, visual acuity in the left eye improved to 20/30. The left eye was orthophoric and there was no proptosis. The conjunctiva looked devoid of any major lymphangioma cysts (Figure 4).

## 3. Discussion

Venous malformations and LMs are common vascular lesions. The Orbital Society created a new classification of vascular lesions based on hemodynamic behavior. The Orbital Society's hemodynamic classification divides vascular malformations into three categories by flow characteristics: no flow (type I), venous flow (type II), and arterial flow (type III) [4]. Capillary hemangiomas and VMs are considered as hamartomas by this classification. Rootman et al. reclassified orbital vascular malformations based on the International Society for the Study of Vascular Anomalies (ISSVA) classification [5]. In this classification, cavernous hemangioma was categorized as a nondistensible cavernous venous malformation. Lymphatic malformations (also known as LMs) were divided into macrocystic, microcystic, or mixed (macrocystic/microcystic) subtypes. Recently, the ISSVA issued a revised classification of vascular anomalies. In this revised classification scheme, hemangioma is categorically listed as a benign vascular tumor and lymphangioma as a lymphatic malformation (International Society for the Study of Vascular Anomalies (ISSVA) classification available at http://www.issva.org/UserFiles/file/ISSVA-Classification-2018.pdf. May 2018).

Orbital VM is the most common benign tumor of the orbit in adults. It is a benign, slowly progressive vascular tumor composed of endothelial-lined spaces surrounded by a fibrous capsule. It most commonly presents in middle-aged adults (ages 20-40 years) and women are affected more than men. Its location is most often within the muscle cone, lateral to the optic nerve. There is no evidence to suggest a heritance pattern in most cases. In our case, the tumor was located in the inferonasal orbit causing slight lateral displacement of the globe [6].

Orbital VM is generally accepted to be a congenital abnormality although it usually becomes symptomatic later in life. Venous malformations are composed of a network of vascular channels separated by fibrous tissue stroma. Histopathologically, VM is composed of dilated, cavernous vascular spaces separated by connective tissue stroma. Flattened endothelial cells line the vascular spaces, which are filled with blood. Single or multiple layers of smooth muscle cells surround the vascular spaces [6, 7].

Lymphatic malformations are multicystic, localized malformations that involve the lymphatic and vascular systems. Their histology ranges from capillary-sized small vessels to macroscopic fluid-filled vessels. Orbital LMs generally have minimal internal blood flow and an absence of connection to the vascular system (type 1 vascular malformation). They are also believed to be congenital similar to cavernous VM. The exact cause of LM formation is unknown, but most cases are believed to be sporadic. LMs affecting the periocular area are usually seen in the orbit. However, isolated conjunctival LM may also rarely occur, as in the current case [7, 8]. Our case also had a presumed LM on the left hard palate but MRI did not reveal any evidence of LM in the orbit and brain.

Anterior segment optical coherence tomography is a noncontact, noninvasive imaging device that provides high-resolution, real-time, and in situ visualization of tissue microstructure [9]. Although the biomicroscopic findings were suggestive of conjunctival LM in our case, AS-SSOCT was helpful in establishing the tentative diagnosis and in the planning of surgery.

The most important lesion to consider in the differential diagnosis of conjunctival LM is conjunctival lymphangiectasia [10]. Conjunctival lymphangiectasia represents approximately <1% of all conjunctival tumors [11]. Our case had a multicystic septated appearance on AS-SSOCT in contrast to the fewer slit-like, slender, fusiform appearing cysts seen in lymphangiectasia [10]. Further, histopathologic examination revealed multiple prominent lymphatic channels and stromal lymphocytic aggregates, findings that would not be normally expected in conjunctival lymphangiectasia [10]. Conjunctival lymphangiectasia contains only scattered inflammatory cells in the stroma and may feature squamous metaplasia and keratinization of the overlying surface epithelium [10].

Orbital VM can coexist with other tumors including adenoid cystic carcinoma and schwannoma in the same orbit [12, 13]. The occurrence of multiple vascular anomalies in the same orbit has also been reported. Examples include VMs associated with varix, lymhangioma, and arteriovenous malformation [14, 15]. Furthermore, VM and LM have been reported to occur together in other body parts including ulnar nerve and lymph nodes [16, 17].

We postulate that the ipsilateral coexistence of conjunctival LM, hard palate LM, and orbital VM as in our case represents an unusual maldevelopment in vascular embryogenesis. The fact that all 3 lesions occurred ipsilaterally may point out a common pathway in pathogenesis. Our search of the PubMed database using the key words “lymphangioma,” “lymphatic malformation,” “conjunctiva,” “orbit,” “cavernous venous malformation,” “venous malformation,” and “cavernous hemangioma” failed to disclose any previous reports demonstrating ipsilateral coexistence of histopathologically confirmed conjunctival LM and orbital VM as well as a presumed LM on the palate.
